# Micropatterned Neurovascular Interface to Mimic the Blood–Brain Barrier’s Neurophysiology and Micromechanical Function: A BBB-on-CHIP Model

**DOI:** 10.3390/cells11182801

**Published:** 2022-09-08

**Authors:** Ajay Vikram Singh, Vaisali Chandrasekar, Peter Laux, Andreas Luch, Sarada Prasad Dakua, Paolo Zamboni, Amruta Shelar, Yin Yang, Vaibhav Pandit, Veronica Tisato, Donato Gemmati

**Affiliations:** 1 Department of Chemical and Product Safety, German Federal Institute for Risk Assessment (BfR), 10589 Berlin, Germany; 2 Department of Surgery, Hamad Medical Corporation (HMC), Doha 3050, Qatar; 3 Department of Vascular Surgery, University of Ferrara, 44121 Ferrara, Italy; 4 Department of Technology, Savitribai Phule Pune University, Pune 411007, India; 5 College of Science and Engineering, Hamad Bin Khalifa University (HBKU), Doha 24404, Qatar; 6 Dynex Technologies, 14340 Sullyfield Circle, Chantilly, VA 20151, USA; 7 Department of Translational Medicine, University of Ferrara, 44121 Ferrara, Italy; 8 Centre Hemostasis & Thrombosis, University of Ferrara, 44121 Ferrara, Italy

**Keywords:** blood–brain barrier, micropatterning, astrocyte, neuropathology, calcium signaling

## Abstract

A hybrid blood–brain barrier (BBB)-on-chip cell culture device is proposed in this study by integrating microcontact printing and perfusion co-culture to facilitate the study of BBB function under high biological fidelity. This is achieved by crosslinking brain extracellular matrix (ECM) proteins to the transwell membrane at the luminal surface and adapting inlet–outlet perfusion on the porous transwell wall. While investigating the anatomical hallmarks of the BBB, tight junction proteins revealed tortuous zonula occludens (ZO-1), and claudin expressions with increased interdigitation in the presence of astrocytes were recorded. Enhanced adherent junctions were also observed. This junctional phenotype reflects in-vivo-like features related to the jamming of cell borders to prevent paracellular transport. Biochemical regulation of BBB function by astrocytes was noted by the transient intracellular calcium effluxes induced into endothelial cells. Geometry-force control of astrocyte–endothelial cell interactions was studied utilizing traction force microscopy (TFM) with fluorescent beads incorporated into a micropatterned polyacrylamide gel (PAG). We observed the directionality and enhanced magnitude in the traction forces in the presence of astrocytes. In the future, we envisage studying transendothelial electrical resistance (TEER) and the effect of chemomechanical stimulations on drug/ligand permeability and transport. The BBB-on-chip model presented in this proposal should serve as an in vitro surrogate to recapitulate the complexities of the native BBB cellular milieus.

## 1. Introduction

Current state-of-the-art blood–brain barrier (BBB) in vitro models utilize transwell assays or microfluidic channels to culture brain cells. While transwells are mostly static cultures, they fail to replicate shear-stress-induced BBB properties in vitro [1]. Biochemical signals and micromechanical traction forces are proven to be essential factors that influence cell differentiation and development via coordinated cell–cell communication [2]. Traction forces and geometrical constraints play a role in influencing the formation of biological tissue and maintaining tissue homeostasis in vivo [3]. It is therefore important to consider these factors in in vitro organ-on-chip paradigms such as the BBB, lung [4], kidney, skin, and gastrointestinal tract [5]. Cells have been observed to align themselves in a phenotype-specific arrangement when micropatterned in the shape of a ring, triangle, bowtie, or line features [6,7]. Additionally, force generation plays a major role in the migration, proliferation, and left/right asymmetric arrangement of cells and provides novel insight into multicellular development in vitro [6]. The structural integrity of the cytoskeleton of the cell is important for normal homeostasis and regeneration. Flow-induced shear stress on endothelial cells’ morphology and function has been widely explored in in vitro BBB studies [8,9]. However, the effect of endothelial cell morphology and alignment without flow-induced stress, as an essential aspect of BBB properties, has recently been identified [10].

With the advent of lithographic techniques in cell culture, there was a corresponding increase in research to understand the effects of confinement on cellular morphology and, in turn, on cellular structure and function [11]. However, there is no comprehensive functional in vitro model that shows how the neurovascular unit at the BBB is influenced by spatiotemporally distributed micromechanical forces in coordination with biochemical signaling. The results from our previous study indicated the asymmetric alignment of micropatterned mammalian cells on appositional ring boundaries when treated with carbon [6]. Though cellular symmetry is a fundamental morphogenesis process, its effect on several basic bioengineering aspects, including cellular migration, wound healing, and tissue growth in unicellular and multicellular systems, is a relatively understudied concept [12,13]. Applying force to a cell will have an impact on the cytoskeleton and, therefore, the normal function of a cell. In this context, the specific location, magnitude, and direction of the forces are key factors in in vitro tissue regenerations.

**Figure 1:**
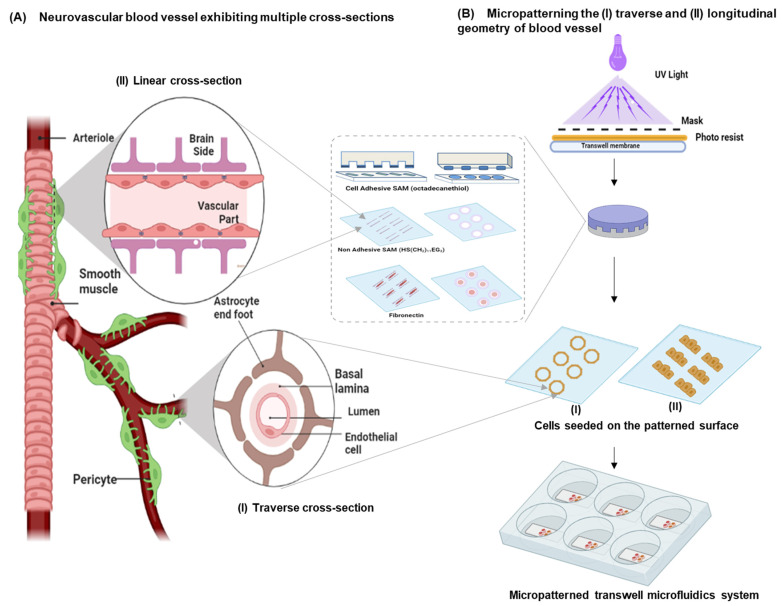
Microprinting of blood vessel architecture on a transwell: (**A**) blood vessel indicating a linear (longitudinal) geometry from the side view and a traverse (ring) geometry from a top view of the neurovascular unit; (**B**) micropatterning by microcontact printing with a cell-adhesive monolayer representing the side view of a traverse and a longitudinal cross-section of the blood vessel. A negative photoresist model was made with UV light through a mask containing the desired micropatterns as ring and linear geometries. PDMS elastomeric stamps were cast with prepolymers and a curing agent on the mold. Cell-adhesive octadecanethiol, a self-assembling monolayer (SAM), was transferred onto the transwell membrane and further subjected to nonadhesive ethylene glycol terminated SAM and fibronectin.

The primary objective of this study was to decode the effects of micropatterning-based endothelial cells as ring and linear geometries to exhibit in-vivo-like BBB features in terms of tight junction expression, paracellular permeability, and overall barrier properties (schematic Figure 1A). The model in this study was adopted to mimic the in vivo polarity (luminal–transluminal) of the neurovascular unit [14]. Additionally, the study aimed to understand the pathological implications of the in vitro blood–brain barrier when exposed to nanoparticles, specifically supramagnetic iron oxide nanoparticles (SPIO-NPs) on development-related cellular functions. The unique in vitro BBB model demonstrated in this report examined (1) the influence of biochemical and collective cell force generation on the BBB phenotype in vitro using human astrocytes and endothelial cells; (2) the robustness and effectiveness of micropatterning transwell inserts to create an in vitro BBB-on-chip model. Additionally, there was the opportunity to map dynamic traction forces involved in regulating cell–cell interactions in vitro by incorporating printed fluorescent beads beneath adherent cells with microscale precision. We hypothesized that patterned cells exhibit directional bias through spatial and temporal changes in the forces exerted on the underlying surface. The results of the integrated micropatterned transwell co-culture with traction force microscopy (TFM) also extend its applications to multiscale regenerative medicine. This surrogate model provides an unprecedented capability to fabricate, directly monitor, and quantitatively analyze in vivo tissue-level physiological functions such as molecular transport and immune responses to bacteria (ongoing study to probe a TFM-based infection model) and nanoparticle-mediated drug delivery across the BBB.

## 2. Materials and Methods

### 2.1. Cell Culture

Human umbilical vein endothelial cells (hUVECs) (PCS-100-013) were from American Type Culture Collection (ATCC), and human astrocytes from Lonza (CC 2565) were cultured as per the manufacturer’s instructions and used for experiments at a passage of 3–5. The hUVECs were cultured in media containing vascular basal medium (ATCC PCS-100-030) and endothelial cell growth kit-BBE (ATCC PCS-100-040) prepared as per the manufacturer’s instructions. Similarly, the astrocytes’ growth medium, AGMTM (CC3186), was used for culturing the astrocytes. The cells were cultured at 37 °C and 5% CO_2_ until they reached 80% confluency, after which they were trypsinized and seeded onto the desired micropatterned surfaces. 

### 2.2. Micropatterning and Cell Seeding on a Transwell Perfusion System

The whole procedure can be grouped into three steps: (a) elastomeric polydimethylsiloxane (PDMS) stamp design: (b) protein microcontact printing; (c) hUVECs’ culture on the cell-adhesive line/ring patterns in step 1, and the initial mold was obtained using a negative SU-8 2050 photoresist (Micro-Chem Corp.) and chromium photo mask containing line and ring geometries by UV exposure. PDMS monomer and Dow Corning curing agent (SYLGARD™ 170 Silicone Elastomer Artikel-Nr.0721SY17010) at a ratio of 10:1 were poured onto the developed mold and cured for 4 h at 70 °C to obtain PDMS stamps. The PDMS stamps were used to transfer the octadecanethiol (Merck Millipore, Berlin, Germany; catalogue number: 814444), as self-assembled monolayers (SAMs), onto the gold-sputtered nanocoating at the transwell membrane. These SAMs acted as surface-active molecules for immobilizing functional anchoring units similar to proteins in the extracellular matrix, such as collagen and fibronectin, in step 2 of the protocol. The transwell membrane was then immersed in ethylene-glycol-terminated SAM (HS-(CH_2_)_11_-EG3, Prochimia) for 3 h, which created cell-adhesive ring/line patterns (ring diameter: 250 µm; line width: 250 µm; aspect ratio: 1:4) from the previous step 1. Before surface sterilization, the inlet and outlet channels were defined by punching holes (diameter: 0.5 mm) under mild force and flushed with ethanol to remove debris. Lastly, the patterned surfaces were surface sterilized using ethanol and incubated with ECM protein, 25 µg/mL fibronectin (Sigma, St. Louis, MO, USA), for 30 min prior to seeding (Figure 1, PDMS stamping on the transwell). With a flow rate of 15.5 mL/min, a shear stress of 637 dynes/cm was reached via applying a target pressure of 20 mbar with syringe pump custom-made with pump control software (ibidi, Leipzig, Germany). Each transwell was equipped with its own inlet–outlet loop, and the perfusion was controlled by a single pump.

In the step 3, the final step, the cultures were seeded in 6-well corning transwell plates with a growth area of 4.5 cm^2^, pore density of 100 × 10^6^/cm^2^, and pore size of 0.4 µm. Inlet–outlet perfusion was created by puncturing the micropatterned transwell surface. Pre-coating of the transwell membrane surface was conducted with 10 mg/mL fibronectin for 30 min. Initially, astrocytes were cultured in the basolateral side of the transwell and allowed to adhere. The hUVECs (seeding density: 1 × 10^5^ cells/well in 6-well culture plates) were cultured on the apical side of the transwell insert. After 24 h, the transwell inserts were placed in 6-well culture plates facing the abluminal surface, where the astrocytes were cultured. The cells were allowed to grow in co-culture under perfused conditions for a total of 72 h (unless mentioned otherwise) before different characterization techniques were used as described subsequently.

#### 2.2.1. Analysis of the Transendothelial Electrical Resistance (TEER) and Impedance Spectroscopy

The transendothelial electrical resistance (TEER) was measured using a Millicell ERS-2 (Electrical resistance system) with an STX2 electrode (Merk, Darmstadt, Germany) to characterize the barrier function of the hUVEC–astrocytes transwell microfluidics [15]. The ERS meter worked by applying an AC-square-wave current and recorded the electrical resistance (*R*) generated in response. A background control of the microprinted membrane coated with fibronectin was also measured (*R_m_*) and subtracted. The TEER value was calculated for the unit area normalization using the cell growth area (*A*) of the transwell microfluidics system with the following formula:

(1) $$TEER=\left(R-R_{m}\right)\times A$$

Prior to the TEER measurements, functionality checks on the Millicell ERS-2 m were conducted by connecting the STX04 test electrode through an inserting plug. The “R Adj” setting on the meter was adjusted until the meter displayed 1000 Ω. Care was taken during the TEER measurements to insert the electrode in such a way that the shorter tip was dipped in the culture plate insert, and the longer tip was immersed in the outer well. It was made sure that the shorter tip did not contact the cells growing on the membrane, and the longer tip only slightly touched the bottom of the outer well. The electrodes were held steady and at a 90° angle to the plate insert. The TEER measurements were performed for the unpatterned hUVEC microfluidic system, patterned with only hUVECs and patterned with co-culture subsequently on days 1, 3, and 7. For the pathology studies with nanoparticle exposure, the microfluidic cell culture system was initially exposed to nanoparticles for 24 h subsequently followed by TEER measurements. To ensure cell survival, the cells were supplemented with fresh media on both the transwell as well as the basolateral well every day until the seventh day of the TEER measurements.

##### L–R Asymmetric Distribution Analysis

We acquired high-resolution phase contrast images of the live patterned hUVECs, with each pixel representing 0.645 μm. Based on the automated detection of the intensity gradient and circular statistics, the L-R asymmetry was quantified using a custom-written code in MATLAB (MathWorks R2021a). A Gaussian differential filter was utilized in the algorithm to determine the intensity gradient pixel by pixel in each image portioned into subregions for analysis. An accumulator scheme in the algorithm was adopted to determine the dominant local direction base on the orientation of each pixel utilizing the von Mises distribution. All subregions were analyzed using circular statistics to determine the mean angle and standard deviation of the biased alignment. More than 50 rings were analyzed for each geometry in at least three independent experiments (*n* = 3). Using the deviation from the circumferential angle of each subregion, the orientation was converted into an angle bias for each subregion.

#### 2.2.2. Tight Junction Visualization by Immunofluorescence

The cells were routinely analyzed for cell phenotype and tight junction (TJ) expression by following immunostaining protocols. In brief, cells were seeded at a density of 5 × 10^4^ cells for hUVECs and 2.4 × 10^4^ for astrocytes. Control samples with no astrocytes were also used. The expression of endothelial junctional proteins (i.e., ZO-1, occluding, and claudin-5) on the endothelial cells was assessed by staining in the absence (0A) and presence (EA) of astrocytes (Figure 1). The von Willebrand factor (vWF) from AbCore Germany (cat no. AC12-0202), glial fibrillary acidic protein (GFAP) from Thermofischer (catalog no. MA5-12023), and GLUT-1 from rnd systems (Catalog no. FAB1418R) were detected by immunocytochemistry as cell-specific markers [16].

The cells were fixed with 4% formaldehyde in cytoskeletal buffer (i.e., 10 mM MES, 138 mM KCl, 3 mM MgCl_2_, 2 mM EGTA, and 0.32 M sucrose) for 30 min. Actin staining for the cytoskeleton was performed using Phalloidin TRITC (1:400; Catalog No. R415 Invitrogen, Berlin, Germany) for 1 h. Nuclei were stained using 4′,6-diamidino-2-phenylindole (DAPI). The phase contrast and fluorescence images were obtained using an LSM 700, and a Zeiss microscope was for the morphology and alignment analyses. Image analysis was conducted by merging the stained actin (red) and DAPI images using ImageJ (NIH) [17]. Confocal and fluorescent microscopy were utilized to visualize and compare the morphology of the hUVECs and co-culture (Figure 1). 

The TJ analysis was performed by immunostaining ZO-1 based on our previously reported protocol for ZO-1 staining [18,19]. For ZO-1 immunostaining, the micropatterned cells on the membrane cells were washed 3 times with phosphate-buffered saline (PBS), and the cells were fixed with 4% paraformaldehyde for 15 min at room temperature. Permeabilization of the samples was conducted with 0.2% Triton X-100 for 10 min. Subsequently, a blocking step with PBS containing 10% fetal calf serum (FCS) was performed. The primary antibody against ZO-1 (clone: ZO-1-1A12, Alexa Fluor 555 conjugate) was added at a ratio of a 1:200 dilution in PBS containing 1% FCS, and the sample was incubated at 4 °C overnight. The secondary antibody was diluted at a ratio of 1:400 in PBS containing 1% FCS and incubated for 1 h at room temperature for staining. Later, the cells were washed with PBS three times and counterstained with Hoechst (1 μg/mL), and the dishes were analyzed by confocal laser scanning microscopy (LSM 700, Zeiss, Berlin, Germany). Microscopic images of the fixed samples were acquired using a 40× or 63× objective.

Images of the micropatterned cells were recorded and analyzed by ImageJ software. The orientation was determined by fitting the cells with an eclipse representation. The degree of polarization was denoted as the ratio of the major to the minor axis, and the orientation was denoted by the angle (θ) between the major axis of the eclipse and the patterned axis ranging from ±90°, where 0° indicates the direction of the underlying line pattern.

##### Probing Micromechanical Forces at the Cell–Cell Interface Using Traction Force Microscopy (TFM)

To conduct the traction force analysis, micropatterned polyacrylamide gels (PAGs) with known mechanical properties were generated. The PAGs were micropatterned by adapting the above procedure. Live cell imaging of the patterned hUVECs cells was carried out in conditioned media (i.e., media collected from 3 day old in vitro cultures of hUVECs and astrocytes) on a patterned membrane as explained in our previous report [20]. The mechanical properties of the polyacrylamide membrane (micropatterned cell culture surface) were generally well characterized and could be controlled by altering the gel composition. For the routine TFM protocol, fluorspheres were incorporated into the PAGs and brought to the surface during gel polymerization and the centrifugation step (15 min at 600 rpm). During the polymerization step, laminin/fibronectin-coated ring micropatterns were transferred to the gel using a stamp. This micropattern provided the cells with a geometrically defined area for adherence. The PAG membrane was placed on the luminal face of the transwell, and the endothelial cells were seeded onto the micropattern and allowed to reach confluence. The transwell inserts were then placed in the 6-well plates containing precultured astrocytes or conditioned cell media. The cells were then imaged once every 10 min for 24 h, using both phase contrast and fluorescent microscopy to excite the fluorophores. This allowed for the visualization of the pull–push traction forces applied to the endothelial cells in the absence and presence of astrocyte-conditioned cell media. The time-lapse images were then analyzed using ImageJ software: particle image velocimetry (PIV) and fast Fourier transform normalized cross-correlation to measure the deformation of the fluorosphere beads. These deformations were translated into forces for further analysis.

##### Calcium Imaging to Study the Astrocyte-Mediated Neurovascular Coupling at the BBB Interface

Calcium imaging is a commonly used measurement of calcium signaling in cells and is generally achieved by using calcium indicator dyes. These dyes change the spectral properties when Ca^2+^ ions are bound, which can be exploited as a measure of calcium signals irrespective of the concentration [21]. Intracellular calcium signaling was monitored by a Calcium Probe Fluo-4 AM. Cells were seeded on the patterned and control PDMS surfaces for 2–4 h; then, 10 μM fluo-4 AM was added to the cell media to dye the cellular calcium [22]. The cell culture media with fluo-4 AM were replaced with fresh media containing 100 µm probenecid to reduce the leakage of indicator and enhance dye loading. Prior to the calcium measurements, the medium was replaced with Hank’s balanced salt solution to minimize the background noise created by the calcium and salt in the cell culture medium. Cells were kept in the dark at room temperature for 20 min for the de-esterification reaction, and Ca^2+^ imaging was performed with a confocal microscope.

Live cell imaging occurred every 3 s using a cooled CCD camera (Zeiss Z-observer). The intracellular calcium concentration ([Ca^2+^]_i_) amplitude was quantified by measuring the average image intensity of the fluo-4-positive cells in all time-lapse fluorescence images. The ImageJ-based plugin, spark master, was used to analyze the calcium oscillations and their morphology [23].

The intracellular calcium concentration ([Ca^2+^]_i_) amplitude was also quantified utilizing a method described previously [24]. The calcium concentration was calculated by [Ca^2+^]_i_ = K_d_ × (F − F_b_)/(F_max_ − F), where F is the fluorescence intensity inside the cell; F_max_ and F_b_ are the fluorescence intensity when saturated with and in the absence of Ca^2+^; K_d_ is the ion dissociation constant, and it was assumed to be 345 nM [25]. The peak fluorescence, F_max_, was obtained by treating the astrocytes with the calcium ionophore.

#### 2.2.3. Statistical Analysis

All experiments were conducted in triplicate (*n* = 3), and the values are reported in the text as the average value ± standard deviation (SD). The Student’s two-tailed *t*-test was used for statistical significance for comparison between groups with normal distributions. The Student’s two-tailed *t*-test was determined and used as reported in our research [26]. Error bars in the figures are either the SD or the standard error of the mean, as indicated in the legends. A *p*-value of <0.05 was considered to be statistically significant.

## 3. Results

### 3.1. Design Considerations

This study was conducted to effectively replicate the microvasculature of the BBB and with the ability to identify the pathophysiological mechanisms of neurodiseases. From a bioengineering perspective, an ideal in vitro replica of the BBB should comprise three major components [27]:

- An array of endothelial cells exhibiting the multicellular longitudinal and radial architecture of blood vessels. This can be achieved by patterning the endothelial cells as a linear (i.e., longitudinal cross-section) or a ring geometry (i.e., similar to the radial cross-section of a blood vessel). Micropatterning can provide mechanistic information on endothelial cell morphology;
- Constant and continuously perfused transwell chambers that simulate the BBB microvessel physiology. By applying a microfluidics-based perfusion system, information on the disease pathology arising from endothelial functions can be identified;
- Astrocytes that wrap around the endothelial capillaries via end-feet processes at the transluminal surface (i.e., away from the blood vessel lumen) and maintain apicobasal polarity at the tissue–tissue interface of this in vitro neurovascular unit.

**Figure 2:**
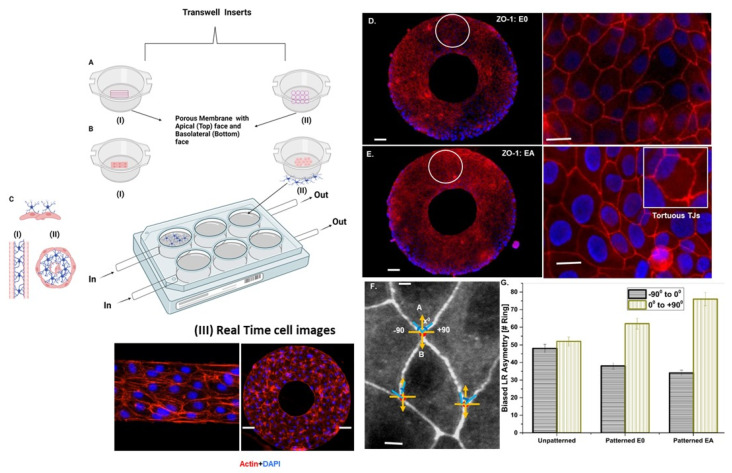
Development and characterization of micropatterned co-culture of HUVECs and astrocytes for in vitro BBB design: (**A**–**C**) Two micropatterns in the form of (**I**) line and (**II**) ring patterns developed on transwells by culturing endothelial cells on line/ring patterns; (**III**) Real time cell images; (**D**–**F**) tortuous and chiral expressions of ZO-1 tight junctions at the BBB from endothelial–astrocyte (EA) co-culture in the contact mode, with the astrocytes cultured at the transluminal surface (**bottom**) of the transwell insert (**B**) versus the astrocytes cultured in the routine culture well (**C**); (**G**) quantification of the L–R asymmetric distribution of the TJs (scale bar: (**D**,**E**) **left** and **right** magnified panels: 50 and 20 µm, respectively; (**F**) 10 µm).

As shown in Figure 2A–C, these three goals were achieved by adopting microcontact printing of cell-adhesive ECM proteins on porous transwells customized with a perfusion culture inlet/outlet and micropatterning the hUVECs. Subsequently, one set of transwells were dedicated to culturing hUVECs that were precultured with astrocytes at the bottom of the cell culture plates to realize the BBB endocytic feet via co-culture (EA). Another two sets were adopted as the control, with endothelial cells in a transwell microcontact-printed monoculture (E0) and an additional transwell unpatterned hUVEC culture. Micropatterning of linear and ring geometries was performed, as shown in Figure 1, to replicate the longitudinal and radial symmetry exhibited by blood vessels. This was confirmed by the linear and radial cross-sections of the blood vessel architecture staining the cytoskeleton actin and nuclei. We also confirmed the high fidelity of the printing ECM proteins on the transwell membrane and the better geometrical control of the cell architecture. Such a design represents a minimalist model with better cell–cell interaction and barrier properties.

### 3.2. Astrocyte-to-Endothelial Interaction Promoted Tortuosity and Planar Cell Shape via Chiral Distribution of Tight Junctions

In order to understand the effect of cell alignment in terms of TJ expression, micropatterning was adapted to patterns cells in linear and radial cross-sections. While TJ expression under co-culture and monoculture has been studied in detail, tight junction expression is a relatively understudied characteristic in terms of micropatterning. It was noted that ZO-1 expression was more prominent in the hUVEC co-culture with astrocytes than in the monoculture of hUVECs (Figure 2D,E). To better understand the influence of micropatterning and co-culturing with astrocytes on TJ expression, alignment studies were conducted for unpatterned endothelial cells (control), patterned endothelial cells without astrocytes culture (E0), and endothelial cells in co-culture with astrocytes (EA). A tight junction primarily has two roles: (I) restricting paracellular transport (gate function) and (II) segregating the apicobasal domains of the cell membrane (function). The higher ZO-1 expression evidenced in the current study in the endothelial–astrocyte co-culture was similar to the results of several studies that identified better barrier properties by TJ expression when co-culture or endothelial culture in astrocyte-conditioned media were used [28,29].

The corresponding fluorescent images of actin staining in the ring patterns and line patterns are shown in Figure 2III. It was identified that the cells aligned along the circumferential direction at the ring edges. Cell alignment in terms of the biased angle between −90° and 90° based on the angle of deviation from the circumferential direction was noted. A negative value represents deviation in the clockwise direction, and a positive value represents alignment in the counterclockwise direction (L–R asymmetry). As can be seen from Figure 2F,G, a clockwise alignment (R–L symmetry) was preferred in comparison to a counterclockwise alignment. Additionally, it was noted that unpatterned hUVECs exhibited poor alignment, whereas co-culturing with astrocytes and micropatterning significantly improved alignment bias towards a left–right asymmetry (*p* < 0.05). Thus, unpatterned hUVECs exhibited no preferential alignment, indicating an almost equal clockwise (−47°) and counterclockwise alignment (+52°). Whereas the patterned hUVEC and astrocyte co-culture preferentially aligned counterclockwise (78°) in comparison with the patterned hUVECs (62°) (Figure 2G).

Further quantifying the TJs using the arc-chord ratio of the looped TJs indicated that they were tortuous and zipped together in co-cultures on the patterned surface. The ZO-1 tortuosity had the ability to enhance interdigitating cell–cell boundaries, which has a role in physical barrier functions. At the boundaries, the tight junctions exhibited L–R asymmetry, mimicking the planar cell-shaped chiral (PCC) domain formation, similar to epithelial morphogenesis. The angle between the ZO-1 distribution at the anteroposterior (AP) and apical boundaries in the unpatterned and patterned hUVECs were quantified using a Python-written custom code (Figure 2D–F). Cell-boundary angles 0 to +90° to the AP axis were more frequent than those of −90° to 0, indicating that patterned transwells induced an LR-biased planar cell shape in the endothelial cells on patterns via hUVECs to astrocytes via apicobasal polar communication (Figure 2). We designated the L–R bias as planar cell-shaped chirality, because the mirror image of the cells’ planar shape did not overlap with its original cell shape.

**Figure 3:**
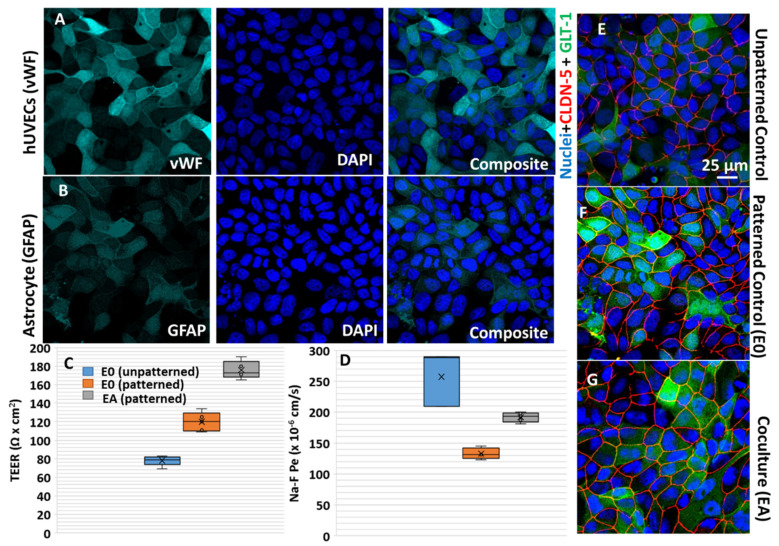
Barrier characterization by immunofluorescence and TEER measurement of hUVECs (patterned and unpatterned), micropatterned endothelial–astrocyte co-culture (EA): (**A**) endothelial cell characterization by expression of factor VIII-related antigen/von Willebrand factor; (**B**) astrocyte characterization was positive for glial fibrillary acidic protein (GFAP); (**C**) TEER for the unpatterned endothelial cells, patterned endothelial cells, and patterned co-culture are indicated in Ω cm^2^ units; (**D**) permeability measurement of sodium fluorescein to determine the endothelial permeability coefficient expressed as Na−F Pe in ×10^−6^ cm/s. Expression of BBB-specific markers, such as glucose transporter 1 (GLUT−1, green) and claudin-5 (CLDN−5, red) counterstained with nuclei stain DAPI (Blue) in blood–brain barrier models constructed for brain endothelial cells under unpatterned (**E**), patterned (**F**), and patterned co-culture conditions (**G**).

The vWF expression is generally used as an endothelial marker and has been identified to be influenced by the microenvironment [30]. Similarly, GFAP has been used for the characterization of astrocyte markers, which is influenced by cell–cell interaction. As can be seen in Figure 3A,B, the growth of astrocyte and endothelial cells can be identified as nonoverlapping and in continuous monolayers. The influence of patterning and co-culture on the expression of primary TJs, such as claudin-5 and ZO-1, and BBB markers, including glucose transporter (Glut-1) and vWF expression, was reported.

Analysis of the morphological characteristics indicated a tightly apposed, elongated, fusiform for endothelial cells, and GFAP immunostaining exhibited differentiated astroglia cells morphology. To effectively study the barrier properties under different culture conditions and to identify the influence of micropatterning and co-culture, immunostaining of TJ claudin-5 and the paracellular permeability of sodium fluorescein (Na-F) was studied. A correlation between the permeability values, transendothelial electrical resistance (TEER), claudin-5, and glucose transporter expression by immunostaining provided the actual effect of the culture conditions on TJ expression and permeability properties. Cellular interaction with astrocytes is widely known to increase TJ expression, as shown in previous studies [31], and it was also confirmed in the present analysis [32]. As can be seen from the overlapping channels in Figure 3E–G and Supplementary Materials Figure S1, with single channel grey scale image for clarity, claudin-5 expression was more prominent in the patterned endothelial–astrocytes co-culture in comparison to the unpatterned and patterned monoculture. This can be attributed to the increase in claudin expression by the activation of sonic hedgehog (Shh), which are primarily released by astrocytes [33]. 

Compared to the monoculture of endothelial cells without patterning in transwell, all patterned models (E0 and EA) showed significantly higher TEER (Student’s *t*-test for the control vs. E0; EA *p*-value of 0.02 and 0.001, respectively, for the TEER). Care was taken to minimize false positive TEER values or discrepancies in the fluorescein assay on the micropatterned surfaces by normalizing with cell-seeding density on the micropatterned area. Micropatterning can enhance the cell junction tightening by 1.5-fold and further the presence of astrocytes in the co-culture more than two-fold in cell tightening (Figure 3C). Corroborating TEER expression, we observed high permeability for the Na-Fluorescein in the control compared to both the patterned monoculture and the co-culture experiments, indicating that the micropatterning and co-culture had a positive influence on the tightness or increased the barrier functions (Figure 3D). Statistical analysis exhibited significantly high permeability for the Na-Fluorescein in the control unpatterned endothelial (E0) vs. patterned endothelial (Student’s *t*-test, *p* = 0.038) monoculture and co-culture (*p* = 0.04, EA). Further, the paracellular permeability of the Na-F was relatively higher in the patterned co-culture conditions relative to the patterned monoculture of endothelial cells.

### 3.3. Endothelial Forces Exhibited Directionality and Enhanced Traction Forces with Astrocyte Co-Cultures

**Figure 4:**
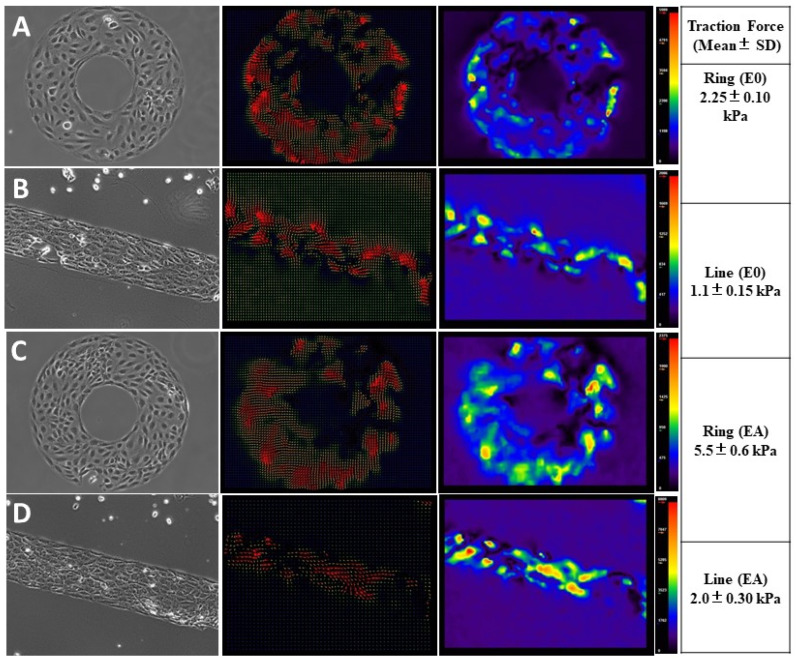
Phase contrast images of hUVECs (left panel) on PAG constrained by microcontact printing (10× magnification). The magnitude and vector map (middle and right panel) displaying the force distribution and direction of the bead displacement on the underlying ring/line micropatterns in the transwells in the absence (**A**,**B**) and presence (**C**,**D**) of astrocyte co-culture (scale bar: 50 µm).

It is experimentally challenging to demonstrate multiscale micromechanical traction forces involved in astrocyte–endothelial interactions at the BBB with defined spatiotemporal cues. In the current study, endothelial cells arranged themselves in a clockwise (CW) orientation on the ring patterns as shown in the previous micropatterning experiments. In the time-lapse imaging, when the cells came into contact with an imposed boundary, they migrated along the barrier edge to the left in a clockwise orientation (Figure 4). To evaluate the intercellular tension, we performed traction force microscopy on a micropatterned BBB model on polyacrylamide gel (PAG). As can be seen in Figure 4, the cells generated patterns of bead displacement towards the center, indicating contractile force generation. Force generation in the cell culture caused a clear pattern of contraction consistent with the migration of the cells. Some forces propelled the cells in a clockwise direction on the outside circumference/edge of the ring–line patterns. This was also the case in the astrocyte co-culture. Interestingly, the magnitudes of the traction forces were amplified in the co-cultures, elucidating the role of astrocyte signaling in the dynamic control over BBB microcirculation. This observation led us to conclude that the tendency to arrange the endothelial cells with a biased left–right asymmetry when micropatterned on rings/lines reflects an asymmetric force distribution in the BBB. This novel micropatterned transwell protocol with a defined apicobasal polarity establishes temporal control of cell–cell interaction and micromechanical regulation of spatial signals. It could be a useful BBB model to predict lead drug compounds or regenerative models in neurobiology.

### 3.4. Astrocytes Regulated Intracellular Calcium Oscillations in Endothelial Cells at the BBB Interface

**Figure 5:**
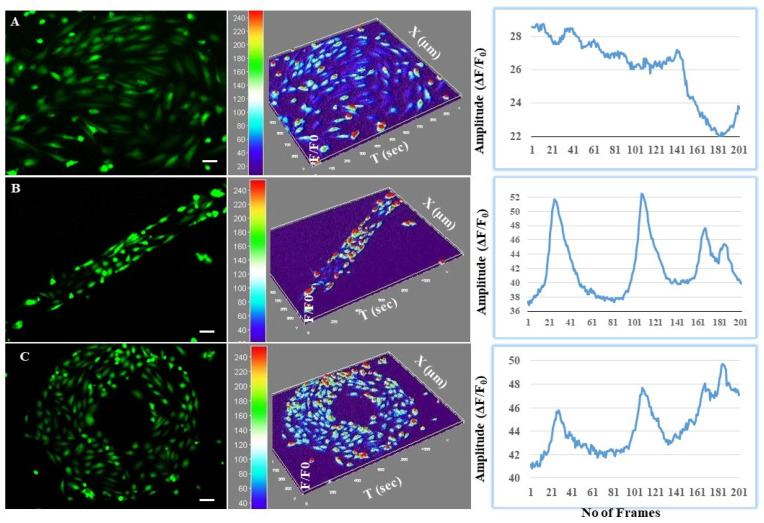
Induced spontaneous calcium sparks in the **upper panel** (**A**) from unpatterned control endothelial cells. The **middle panel** shows the patterned endothelial–astrocyte co-culture (**B**) line patterns and the **lower panel** (**C**) the ring patterns (scale bar: 50 µm). The **left column** exhibits spontaneous calcium release events stained with Fluo-4-AM, the **middle column** shows the average intensity-based pseudo-colored topographical map showing the calcium level in individual cells, and the **right column** shows the ImageJ sparkmaster-extracted intensity features of the calcium sparks to plot the quantitative curves for control unpatterned vs. patterned co-cultures.

To examine how astrocyte and endothelial communication influenced the interplay between traction forces and [Ca^2+^]_i_ signaling at the microfluidics BBB, calcium signaling was measured in astrocytes with a fluo-4 AM calcium probe. In co-cultures (EA), spontaneous calcium sparks were observed analogous to that excitation–contraction (Supplementary Materials Movie S1) [34]. Analysis of the peak morphology revealed significant differences in intensity, oscillation amplitude, time to peak, relaxation time to peak, and the time interval between two neighboring peaks (Figure 5). Intracellular calcium levels displayed more distinct oscillatory responses in the patterned co-culture, indicating stress-induced junctional/barrier properties. Oscillatory [Ca^2+^]_i_ activity was not distinct in the unpatterned cells, while a wave of activity was more prominent (Supplementary Materials supplementary Movie S1). This can be understood better in correlation with the paracellular permeability studied in the previous sections. Higher oscillatory responses indicate an increased TJ expression, resulting in reduced paracellular permeability. 

### 3.5. Perspective of Modeling Proof of Principle Neurodegenerative Disease Pathophysiology on the BBB-on-Chip

Concerning the translation of the new approach methods for neurotoxicity paradigm testing, we performed iron oxide nanoparticles treated perfusion culture as proof of principle disease pathophysiology exploration of the designed chip. In neurological disorders, such as multiple sclerosis (MS), metal and metal oxide (particularly redox-active elements, such as iron copper, zinc, and manganese) overload is well established in contributing to the disease pathophysiology [35,36] as has also been demonstrated in other metal-driven complex diseases [37]. Keeping this in mind, we treated the cultures under perfusion with superparamagnetic iron oxide nanoparticles (SPIO-NPs). A control with no treatment was compared with the results of exposing co-culture or monoculture as shown in the Supplementary Materials Figures S2–S5. It was identified that a reduced tight junction expression of ZO-1 in immunostaining (Supplementary Figure S2A–C) in iron-exposed samples compared to the unexposed controlled. The quantification of the hUVEC barrier functions via cell impedance measurements of the TEER supports the qualitative microscopic data (Supplementary Figure S3). Further, a detailed analysis of calcium signaling perturbation in lieu of exploring the toxicant effects in neurobiology exploration was performed. As shown in the Supplementary Materials Figures S4 and S5, we can see the mitigation of calcium signaling parameters, such as the amplitude; FWHM, full width at half-maximum amplitude; FDHM, full duration at half-maximum amplitude, as analyzed with ImageJ spark master [38]. We can observe that these individual spark parameters were different in the untreated control vs. the treated monoculture (E0) or co-culture (EA), as seen in the histograms and their density-derived quantification, as shown in Supplementary Materials Figure S4. In the control, the amplitude and peak intensity of the calcium peaks were higher, and the time-to-peak and relaxation time were shorter compared to the SPIO-NP-treated samples. Furthermore, additional events were found to be typically narrow (small FWHM and small full width), exhibiting a small full duration but a similar FDHM and peak time in between treated groups. The maximum steepness of the spark upstroke was a measure for the maximal sarcoplasmic reticulum (SR) Ca-release flux underlying the spark, and the time to the peak can be used as a measure for the duration of the Ca release. The exponential time constant of the spark decay, on the other hand, provides insight into Ca buffering as well as SR Ca uptake.

## 4. Discussion

We developed a unique BBB model that aimed to mimic three crucial aspects of actual BBB vasculature viz. shear-induced barrier properties, co-culture based barrier properties, and traverse and longitudinal cross-sections of vessels. This allowed for the realistic investigation of tight junction expression. Moreover, the developed model provided a platform for studying the influence of micromechanical forces on the biochemical responses in an actual BBB environment. Typically, the reconstitution of actual BBB vasculature with respective micromechanical forces and multicellular environment is a challenging feat. To this end, the model proposed in our study easily integrated a perfused system with micropatterned transwells, thereby replicating the flow-induced stress and cross-sectional effects of BBB cells on the barrier properties. It was expected that cellular interactions and communication from an endothelial–astrocyte co-culture would benefit the secretion of adhesion molecules and junctional protein expression, thus maintaining better barrier properties with improved cell growth and survival. This can be attributed to the synergy among the different cell types as mentioned previously in the literature [39]. Similarly, crosstalk between neurons, astrocytes, and endothelial cells has been proven to promote neuronal cell growth [40]. While other co-culture and microfluidic models have been proposed, our model is relatively complex considering the feasibility of studying the effect of micromechanical forces on biochemical signals. The method developed in this study via a micropatterning protocol will complement the existing BBB perfusion system, which enables the microcontact printing of proteins to create BBB specific multicellular patterns on transwells. This method has certain advantages that we understand can influence the geometry shape of the biochemical signaling, on one hand, and the 2D micropatterning protocol can be adopted to print the cellular patterns on soft substrates such as polyacrylamide gel embedded with fluorescent beads, on the other hand. Such soft substrates can be very useful for developing quantitative methods for measuring traction forces from different cells involved in neurovascular end-feet development.

Cellular alignment in BBB is critical for several functions including enhanced barrier properties, secretion of junctional molecules with adhesion properties, nucleus gene expression of tight junctions, and ECM remodeling. Reports suggest that the spatial orientation of cells plays a crucial role in pattern formation in embryogenesis and neuron maturation [41,42]. For instance, unidirectional cell alignment results in the coordinated contractile forces by the tissues of the cardiac system [6]. Similarly, cell alignment in the BBB is crucial for the generation of contractile forces for highly specific barrier properties. The current study demonstrated the feasibility of conducting cell alignment studies to represent the longitudinal and traverse cross-sections of the BBB vasculature and provided an in vitro BBB platform with realistic properties. Studies on the effect of micropatterning in the longitudinal and traverse cross-sections indicated the preferential alignment of the BBB endothelial cells towards a counterclockwise alignment. This was similar to the results of Wan and colleagues, where hUVECs exhibited a counterclockwise alignment in the inner-ring boundary when cultured in a ring pattern [42]. However, the effect of astrocyte co-culture was not taken into account. The role of endothelial cell orientation and alignment in response to micropatterning and flow-induced shear is a relatively understudied parameter in in vitro BBB models. While the effect of shear stress or micropatterning has been studied individually [27,43], the effect of co-culturing with astrocytes and micropatterning studied here can throw more light on the complex mechanisms involved in cell–cell interactions. The current study also demonstrated the specific alignment of cells in co-culture conditions contrary to the monocultures (Figure 2G). For instance, it was identified using a high throughput device that stimulation with cellular strain on hUVECs, a higher degree of anisotropy was noted. However, stimulation with both shear stress and strain, a cutoff value of shear stress was found to influence the cellular orientation [43]. Thus, this model can also be used as a high throughput device to study multiple factors influencing cellular orientation and migration.

Further characterization of the BBB properties using conventional endothelial and astrocytes marker indicated a differentiated but continuous layer of the barrier. Studies on the permeability and transport across BBB are a required part of BBB characterization for any in vitro model. Typical resistance offered by BBB in vivo can be studied effectively by co-culturing BBB relevant cells like endothelium and astrocytes as demonstrated in the current study. The existing in vitro BBB models are commonly assessed by the resistance offered by the cells for transport or permeability of molecules. However, the barrier properties contributed by micropatterning can shed light on the importance and relevance of BBB architecture towards resistance. The high permeability of Na-fluorescein in control (unpatterned) noticed in the current study can be considered as a poor representation of BBB in vitro. Interestingly, the paracellular permeability of Na-F was relatively higher in patterned co-culture conditions relative to the patterned monoculture of endothelial cells which is due to the higher expression of transport proteins that are used for the transport of bioactive compounds across BBB. A decrease/increase in Na-F transport across the BBB depends on organic anion transporter (OAT)-3 and multidrug resistance protein (MRP)-2 expression [44], and further investigation related to the expression pattern of these proteins will add to these results. Transport across the BBB can be achieved by several mechanisms [1], which could result in the paradox of high TEER with relatively high permeability [45]. There has been an ongoing debate on the exact BBB properties that provide the appropriate BBB functions. While junctional molecules are known for sealing the space between endothelial cells, transporter expression can increase the permeability [33]. The co-culture micropatterned endothelial cells further exhibit BBB-specific higher relative expression of tight junction claudin (CLDN) protein and the endothelial markers glucose transporter 1 in response to shear stress as shown in Figure 3E–G. Increased expression of glucose transporter, a phenomenon identified prominently in co-culture conditions [46] has been identified as a contributing factor to higher permeability of several bioactives [44].

Astrocytes form contact with microvessels and the synapses between neurons in vivo; this is important for matching oxygen and glucose transport to neural activity through regulation of local blood flow [47]. While co-culture with astrocytes has been associated with higher TJ expression and cellular interaction, short-term interactions involving receptor-mediated signaling as intracellular calcium waves are another common characteristic of endothelial astrocytes interaction [46]. Intracellular calcium [Ca^2+^]_i_ is involved in the regulation of blood flow; this is supported by the elevation of [Ca^2+^]_i_ at the astrocyte end-feet when neurons are stimulated. However, the role of calcium signaling at BBB ‘neurovascular units’ has not been systematically explored in relation to their linear or radial architecture [48]. Transformed traction forces could be mechanistically associated with calcium signaling via astrocyte-mediated force regulation on patterned endothelial geometry. The influence of micropatterns on the endothelial astrocyte communication was studied by visualising the calcium sparks for linear and radial patterns of BBB. Significant differences in the calcium signalling were noted for patterned, unpatterned and co-culture patterned BBB (Figure 5). In general signal transduction pathways within the endothelial architecture are responsible for barrier opening by some molecules. This process is associated with an increase in the [Ca^2+^]_i_ which is further linked with the tight junction opening [46]. It is commonly acknowledged that Ca^2+^ signals do not appear as a one-time event restricted in time and space, but often occur as repetitive, oscillatory [Ca^2+^]_i_ changes that are also known to spatially spread across boundaries as intercellular calcium waves [49]. Amplitude and peak intensity was higher with short time-to-peak and relaxation time was significantly less on patterned surfaces. We hypothesize that [Ca^2+^]_i_ signaling could be centrally involved in the dynamic force control exercised by astrocytes in the regulation of barrier morphology via astrocyte-to-endothelial communication. We also observed enhanced cadherin expression in patterned cells. Occludin and cadherin expression is dependent on calcium activity [49] and is bound to cell-adhesion molecules that form junctional complexes such as adherent junctions and desmosomes. In the presence of calcium, the activation energy required to establish bonds is minimized. There is also an additional role for occludin and cadherin in modulating migration [50] and allowing cell-cell cohesion and developing migration polarity via mechanotransduction on constrained surfaces [51]. However, this needs further experimentation to elucidate the connection between calcium signaling and cadherin expression observed herein on in vitro-BBB-chip platform. The unique BBB in vitro model demonstrated in the current study exhibits the feasibility of conducting several translational applications in terms of neuro therapy. While the improved barrier properties under micropatterned and perfused conditions can be a platform for high throughput screening, studies with nanoparticle treatment further elucidate the model’s applicability as a toxicity testing platform. Additionally, the specific release of junctional molecules as an effect of micromechanical forces demonstrated by micropatterning and contractile studies proves the model’s capacity for pathological studies as well. 

## 5. Conclusions

In the present study, we successfully developed a hybrid BBB model by micropatterning with ECM proteins and perfusion system integration into a transwell. The difference in barrier properties for the unpatterned monoculture and patterned mono- and co-culture were studied. Micropatterning demonstrated that mechanical stresses play a major role in determining the cellular microenvironment and in the integrity of the barrier properties. Micropatterning further influenced the elongation and orientation of the endothelial cells as an alternate mechanism of stress-induced TJ expression. This provides knowledge on the ability of endothelial cells to respond to stress created by spatial confinement in addition to shear forces. Additionally, this study further adds to the understanding that co-culture with astrocytes improves the barrier properties as well as calcium signaling. Further, the developed BBB model was characterized with desirable BBB-like properties, thus mimicking the in vivo characteristics of appropriate traction forces and calcium signaling. The model also provides a platform to study disease pathology by iron oxide-induced MS-like pathological characteristics in terms of TJ expression and calcium signaling. We envision that this model can pave the way toward high-throughput screening and pathological studies for a variety of neurovascular disorders.

## Data Availability

The raw/processed data required to reproduce these findings cannot be shared at this time as the data also form part of an ongoing study.

## Supplementary Materials

The following supporting information can be downloaded at: https://www.mdpi.com/article/10.3390/cells11182801/s1, Figure S1: Expression of BBB-specific markers such as Claudin-5 CLDN-5 as shown in grey scale for control (unpatterned monoculture on left), patterned (monoculture in middle) and patterned coculture (right); Figure S2: Perspective of modelling proof of principle neurodegenerative disease pathophysiology on blood brain barrier on chip exhibiting tight junction zona occludens-1 (red), glucose transporter 1 (green) and nuclei stained with DAPI (blue) in untreated control (A), treated monoculture endothelial E0 (B) and treated co-culture astrocyte-endothelial EA (C). (D,E) show separate channel of ZO-1 staining for clarity of tight junction expression. Qualitatively tight junction are well expressed in healthy unexposed endothelial cells in left panel, while they are severely compromised in middle panel with monoculture and moderately expressed in coculture exposed with SPIONs but less than healthy cells in left panel.; Figure S3: Quantitative impedance measurement in cell exposed to SPIONs to compare with healthy control cells for TEER evaluation; Figure S4: Analysis of Ca sparks in endothelia controls with and without astrocyte exposed to SPIONs. The histograms of individual spark properties such Amplitude, FDHM, FWHM, TtP, full duration and full width analysis are shown in upper panel (A) control without SPIONs exposure, middle panel (B) SPIONs exposure in monoculture (E0), lower panel (C) Coculture exposed with SPIONs (EA). Histograms analysis is based on 2070 events recorded in 75 images using a Criteria of 3.8 explained in reference1. Plots of calcium sparks were averaged over 5pixels; Figure S5: Spatiotemporal density analysis of Ca sparks in endothelia controls with and without astrocyte exposed to SPIONs. The histograms of individual spark properties such Amplitude, FDHM, FWHM, TtP, full duration and full width analysis are shown in upper panel (A) control without SPIONs exposure, middle panel (B) SPIONs exposure in monoculture (E0), lower panel (C) Coculture exposed with SPIONs (EA). Histograms analysis is based on 2070 events recorded in 75 images using a *Criteria* of 3.8 explained in [38]. Plots of calcium sparks were averaged over 5pixels; Movie S1: Calcium releasing events of the endothelial cells immediately after seeded onto unpatterned (left panel), patterned ring ECM protein (middle panel) and patterned line ECM protein on transwell bottom. The time lapse video was taken at a rate of 3 seconds per frame (FpS).
