# Supplementary material for: Micropatterned Neurovascular Interface to Mimic the Blood–Brain Barrier’s Neurophysiology and Micromechanical Function: A BBB-on-CHIP Model
Source: Cells. 2022 Sep 8;11(18):2801. doi: 10.3390/cells11182801 (PMC9497163; doi:10.3390/cells11182801)
Supplement: Supplementary file 1 [file cells-11-02801-s001.zip › cells-1880647-supplementary.pdf]

Micropatterned neurovascular interface to mimic the blood-brain barrier neurophysiology and micromechanical function: A BBB-on-CHIP model

Ajay Vikram Singh 1,,†, Vaisali Chandrasekar 2,†, Peter Laux 1, Andreas Luch 1, Sarada Prasad Dakua 2,*, Paolo Zamboni 3, Amruta Shelar 4, Yin Yang 5, Vaibhav Pandit 6, Veronica Tisato 7,8 and Donato Gemmati 7,8*

1 Department of Chemical and Product Safety, German Federal Institute for Risk Assessment (BfR), 10589 Berlin, Germany

2 Department of Surgery, Hamad Medical Corporation (HMC), 3050 Doha, Qatar

3 Department of Vascular Surgery, University of Ferrara, 44121 Ferrara, Italy

4 Department of Technology, Savitribai Phule Pune University, Pune 411007, India

5 College of Science and Engineering, Hamad Bin Khalifa University (HBKU), 24404 Doha, Qatar

6 Dynex Technologies, 14340 Sullyfield Circle, Chantilly, VA 20151, USA

7 Department of Translational Medicine, University of Ferrara, 44121 Ferrara, Italy

8 Centre Hemostasis & Thrombosis, University of Ferrara, 44121 Ferrara, Italy

* Correspondence: ajay-vikram.singh@bfr.bund.de (A.V.S.); sdakua@hamad.qa (S.P.D.)

† These authors contributed equally to this work.

Supporting Figure/movie captions

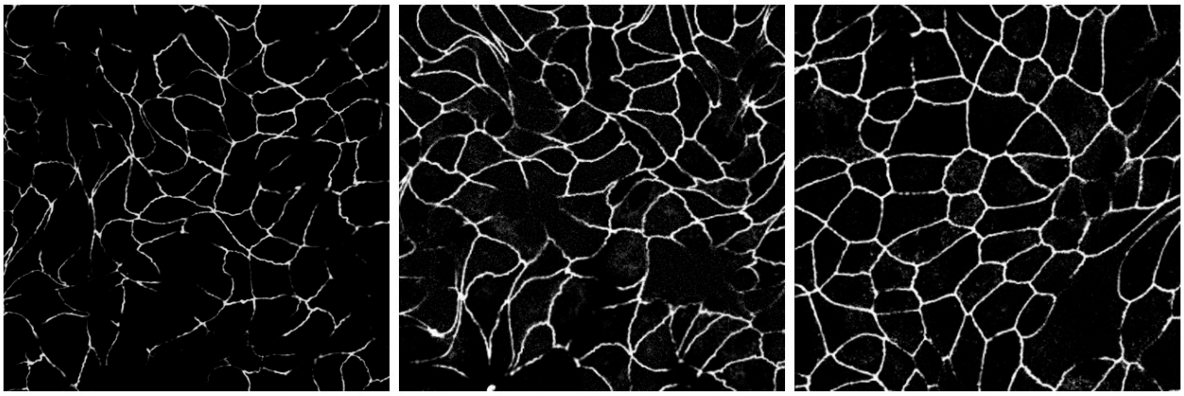

Figure S1. Expression of BBB-specific markers such as Claudin-5 CLDN-5 as shown in grey scale for control (unpatterned monoculture on left), patterned (monoculture in middle) and patterned coculture (right).

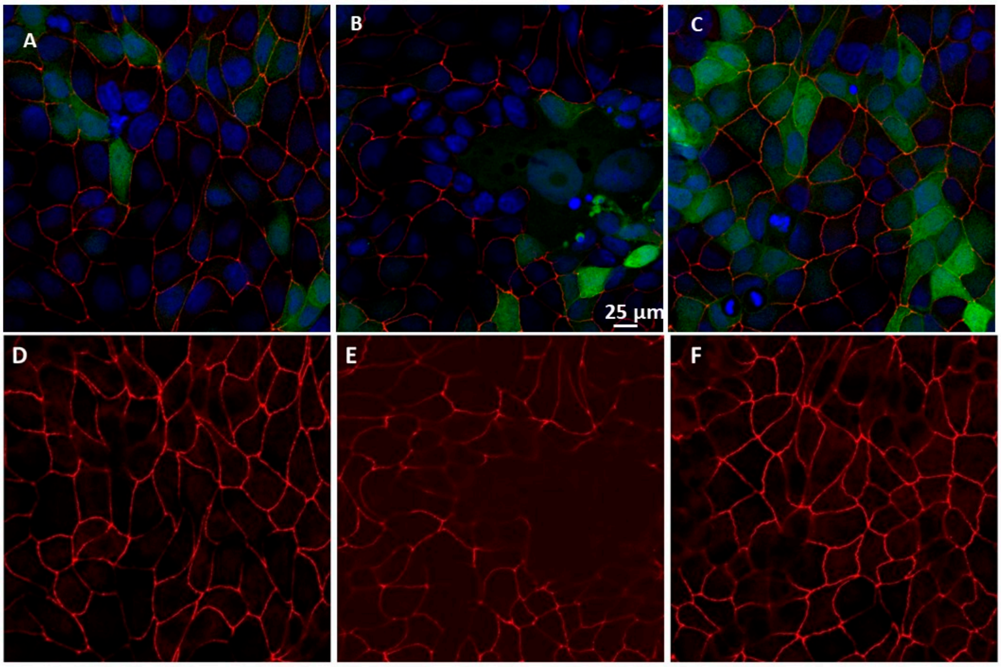

Figure S2. Perspective of modelling proof of principle neurodegenerative disease pathophysiology on blood brain barrier on chip exhibiting tight junction zona occludens-1 (red), glucose transporter 1 (green) and nuclei stained with DAPI (blue) in untreated control (A), treated monoculture endothelial E0 (B) and treated co-culture astrocyte-endothelial EA (C). (D-E) show separate channel of ZO-1 staining for clarity of tight junction expression. Qualitatively tight junction are well expressed in healthy unexposed endothelial cells in left panel, while they are severely compromised in middle panel with monoculture and moderately expressed in coculture exposed with SPIONs but less than healthy cells in left panel.

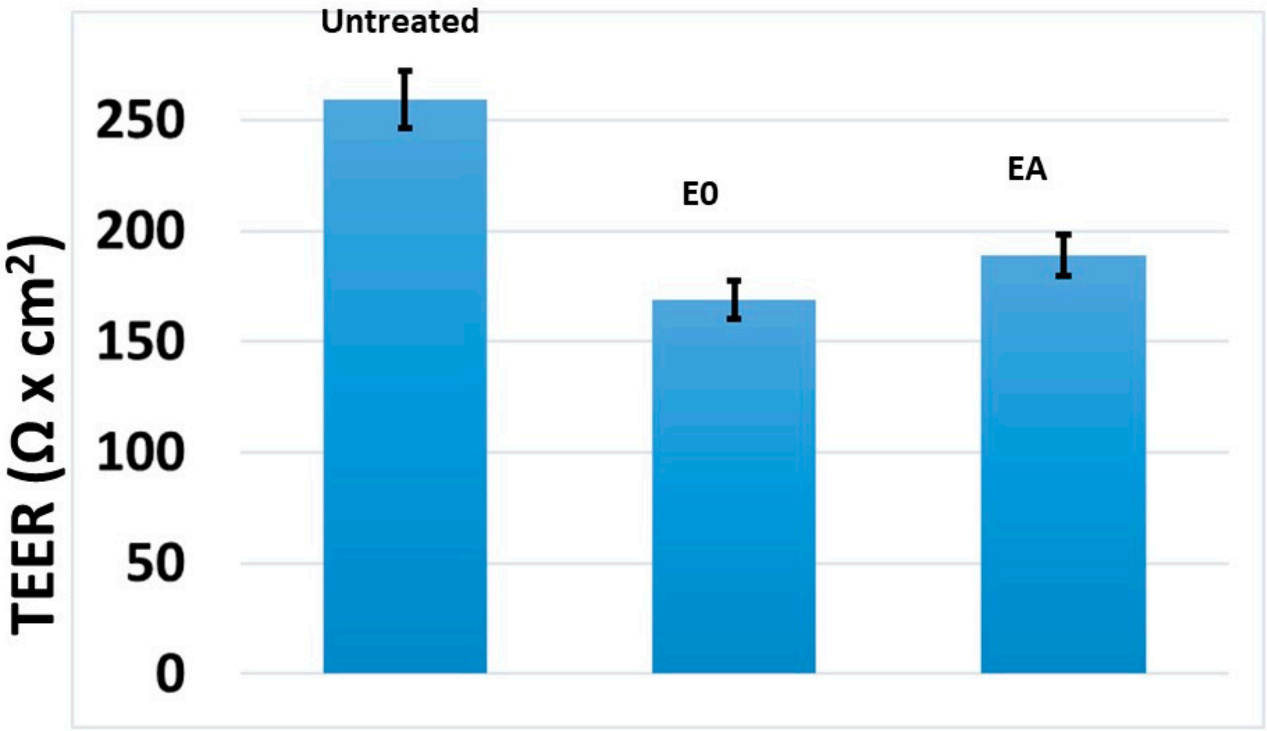

Figure S3. Quantitative impedance measurement in cell exposed to SPIONs to compare with healthy control cells for TEER evaluation.

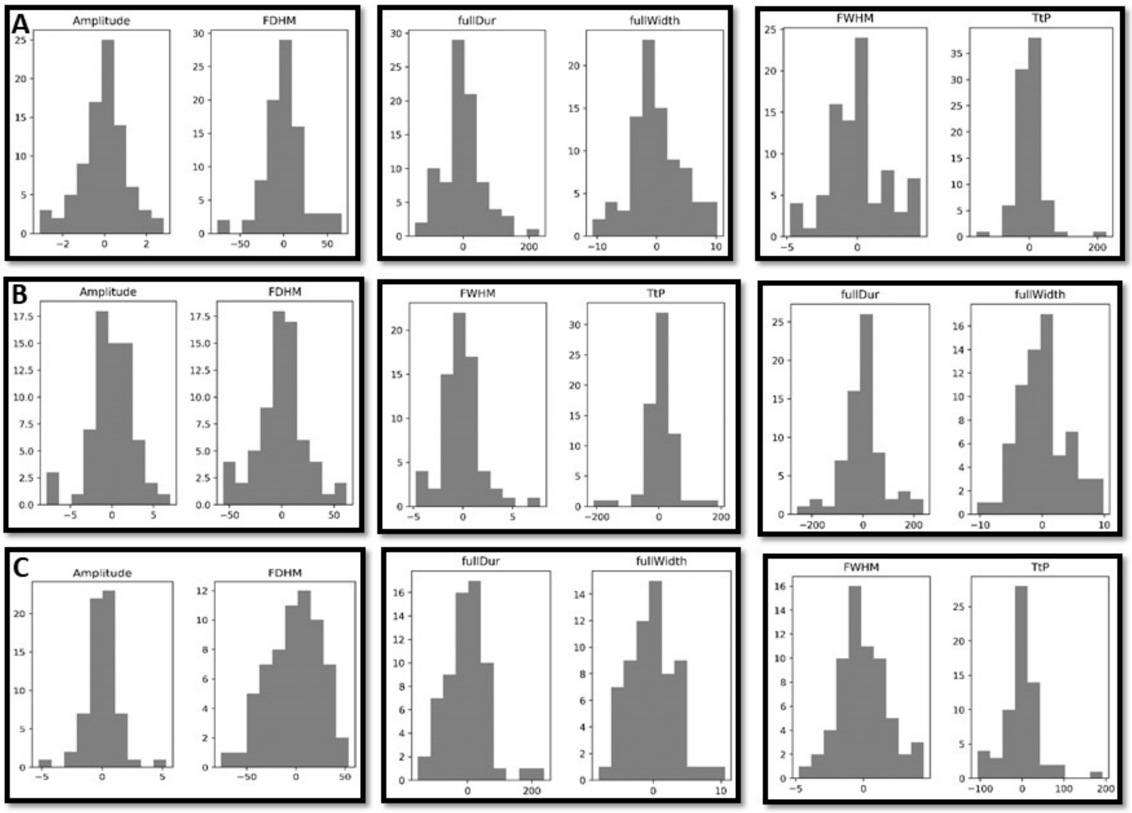

Figure S4. Analysis of Ca sparks in endothelia controls with and without astrocyte exposed to SPIONs. The histograms of individual spark properties such Amplitude, FDHM, FWHM, TtP, full duration and full width analysis are shown in upper panel (A) control without SPIONs exposure, middle panel (B) SPIONs exposure in monoculture (E0), lower panel (C) Coculture exposed with SPIONs (EA). Histograms analysis is based on 2070 events recorded in 75 images using a *Criteria* of 3.8 explained in reference[1]. Plots of calcium *sparks were* averaged over 5pixels.

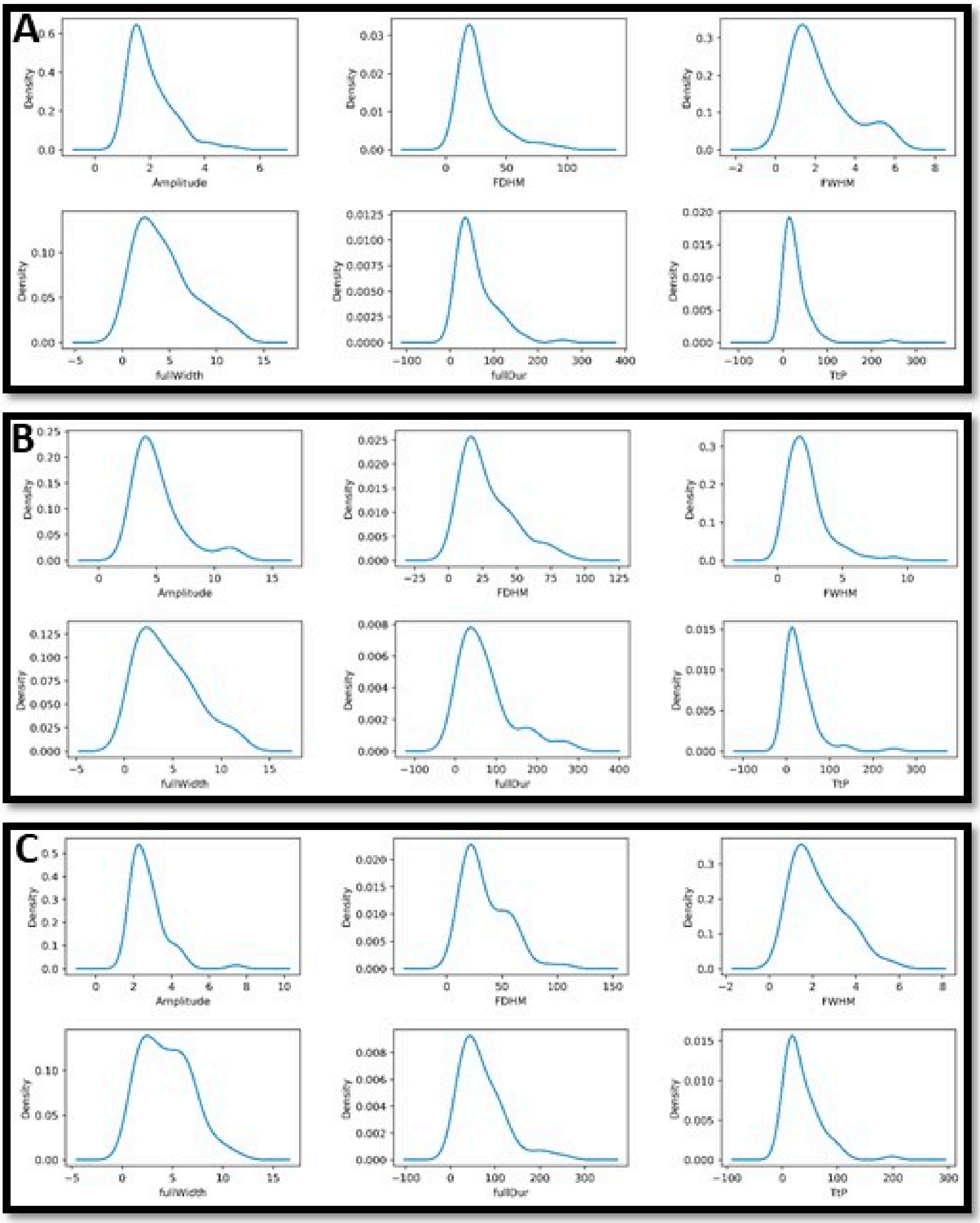

Figure S5. Spatiotemporal density analysis of Ca sparks in endothelia controls with and without astrocyte exposed to SPIONs. The histograms of individual spark properties such Amplitude, FDHM, FWHM, TtP, full duration and full width analysis are shown in upper panel (A) control without SPIONs exposure, middle panel (B) SPIONs exposure in monoculture (E0), lower panel (C) Coculture exposed with SPIONs (EA). Histograms analysis is based on 2070 events recorded in 75 images using a *Criteria* of 3.8 explained in reference [38]. Plots of calcium *sparks were* averaged over 5pixels.

Supporting movie SM1. Calcium releasing events of the endothelial cells immediately after seeded onto unpatterned (left panel), patterned ring ECM protein (middle panel) and patterned line ECM protein on transwell bottom. The time lapse video was taken at a rate of 3 seconds per frame (FpS).
